# Improved Prediction of Survival Outcomes Using Residual Cancer Burden in Combination With Ki-67 in Breast Cancer Patients Underwent Neoadjuvant Chemotherapy

**DOI:** 10.3389/fonc.2022.903372

**Published:** 2022-06-07

**Authors:** Ji-Yeon Kim, Jung Min Oh, Se Kyung Lee, Jonghan Yu, Jeong Eon Lee, Seok Won Kim, Seok Jin Nam, Yeon Hee Park, Jin Seok Ahn, Kyunga Kim, Young-Hyuck Im

**Affiliations:** ^1^ Division of Hematology–Oncology, Department of Medicine, Samsung Medical Center, Sungkyunkwan University School of Medicine, Seoul, South Korea; ^2^ Biomedical Research Institute, Samsung Medical Center, Sungkyunkwan University School of Medicine, Seoul, South Korea; ^3^ Department of Surgery, Samsung Medical Center, Sungkyunkwan University School of Medicine, Seoul, South Korea; ^4^ Department of Health Sciences and Technology, Samsung Advanced Institute for Health Sciences & Technology, Sungkyunkwan University, Seoul, South Korea; ^5^ Department of Data Convergence and Future Medicine, Samsung Medical Center, Sungkyunkwan University School of Medicine, Seoul, South Korea; ^6^ Department of Digital Health, Samsung Advanced Institute for Health Sciences & Technology, Sungkyunkwan University, Seoul, South Korea; ^7^ Biomedical Statistics Center, Research Institute for Future Medicine, Samsung Medical Center, Seoul, South Korea

**Keywords:** neoadjuvant chemotherapy, Ki-67, residual cancer burden, prediction model, breast cancer, residual proliferative cancer burden

## Abstract

We developed a model for improving the prediction of survival outcome using postoperative Ki-67 value in combination with residual cancer burden (RCB) in patients with breast cancer (BC) who underwent neoadjuvant chemotherapy (NAC). We analyzed the data from BC patients who underwent NAC between 2010 and 2019 at Samsung Medical Center and developed our residual proliferative cancer burden (RPCB) model using semi-quantitative Ki-67 value and RCB class. The Cox proportional hazard model was used to develop our RPCB model according to disease free survival (DFS) and overall survival (OS). In total, 1,959 patients were included in this analysis. Of 1,959 patients, 905 patients were excluded due to RCB class 0, and 32 were due to a lack of Ki-67 data. Finally, an RPCB model was developed using data from 1,022 patients. The RPCB score was calculated for DFS and OS outcomes, respectively (RPCB-DFS and RPCB-OS). For further survival analysis, we divided the population into 3 classes according to the RPCB score. In the prediction of DFS, C-indices were 0.751 vs 0.670 and time-dependent areas under the receiver operating characteristic curves (AUCs) at 3-year were 0.740 vs 0.669 for RPCB-DFS and RCB models, respectively. In the prediction of OS, C-indices were 0.819 vs 0.720 and time-dependent AUCs at 3-year were 0.875 vs 0.747 for RPCB-OS and RCB models, respectively. The RPCB model developed using RCB class and semi-quantitative Ki-67 had superior predictive value for DFS and OS compared with that of RCB class. This prediction model could provide the basis to decide risk-stratified treatment plan for BC patients who had residual disease after NAC.

## Introduction

Neoadjuvant chemotherapy (NAC) is a standard therapeutic strategy for patients with locally advanced breast cancer (BC) (1). NAC can reduce tumor burden and downsize tumor mass, resulting in increasing the possibility of breast conservation and avoiding axillary dissection and rendering inoperable tumors operable ones (2–5). More importantly, NAC provides the rationale for de-escalation of surgery in both the breast and axilla and for risk-stratified treatment after curative surgery in BC patients who undergo NAC (1, 6, 7).

A large number of studies have shown that patients who achieve a pathological complete response (pCR) to NAC in both primary breast tissue and ipsilateral axillary lymph nodes have significantly longer overall survival (OS) and disease-free survival (DFS) (8). Additionally, residual cancer burden (RCB) was a significant long-term predictor of DFS and OS in all subtypes of BC (9, 10). In the I-SPY 2 trial, it was suggested that the RCB score could be used to assess the outcomes of novel agents in combination with a standard NAC backbone (11, 12).

However, some patients who achieved pCR have undergone BC recurrence, and the others with RCB III class have had long DFS and OS.

Ki-67 is a nuclear protein expressed in the G1, S, and G2 phases of the cell cycle and not in the resting G0 phase, and it is one of the proliferation markers in many cancers (13). High expression of Ki-67 in tumor cells is associated with tumor growth, higher tumor grades, and poorer survival in breast cancer. Since Ki-67 indicates tumor biology such as tumor growth activity, assessment of Ki-67 can be used to estimate the tumor response to therapies that specifically target dividing cells, such as chemotherapy in particular (14).

Several studies have evaluated the prognostic and/or predictive values of Ki-67. According to these previous studies, a high pCR rate was associated with a high Ki-67 level with statistical significance (15). Additionally, the post-chemotherapeutic Ki-67 value was a strong predictor of survival for BC patients not achieving a pCR (16).

We hypothesized the combination of the biological index of Ki67 with the anatomic index of RCB would provide more prognostic information than either alone. In this study, we developed a prognostic model of BC treated with NAC using an anatomic index of RCB in combination with a biological index of Ki-67. We evaluated the prognostic values of the clinical and pathological characteristics of BC at baseline and curative surgery and made a prognostic model that cooperated with these clinical factors.

## Methods

### Patients

We retrospectively analyzed BC patients diagnosed with clinical stages II to IIIC BC who underwent NAC followed by curative surgery at the Samsung Medical Center between January 2010 and December 2019. Among these patients, patients who received the second operation for ipsilateral or contralateral BC due to local recurrence after initial curative surgery or palliative operation with stage IV disease were excluded. We also excluded patients who underwent surgery for bilateral BC. This study was reviewed and approved by the Institutional Review Board (IRB) of Samsung Medical Center, Seoul, Korea (IRB No. 2019-04-021) with an informed consent waiver due to the use of medical records with retrospective clinical data. This study was conducted in accordance with the Declaration of Helsinki.

### Breast Cancer Pathology

All pathological specimens were reviewed by experienced pathologists who determined primary tumor characteristics based on biopsy specimens obtained for BC diagnosis. Pathologists determined BC histology and receptor status (estrogen receptor [ER], progesterone receptor [PgR], and human epidermal growth factor receptor-2 [HER2]) according to hematoxylin and eosin (H&E) and immunohistochemical (IHC) staining (17). In terms of Ki-67, pathologists assessed it by IHC on the Ventana Discovery autostainer using the antibody MIB-1 as previously described (18). For semiquantitative analysis, signals for Ki-67 were graded by two expert pathologists as follows: 0–25%, 1+; more than 25–50%, 2+; more than 50–75%, 3+; more than 75%, 4+. Histologic grade and nuclear grade were also evaluated by Bloom–Ricardson grading and the World Health Organization grading system (19).

Pathologists determined the pathological response to NAC using surgical specimens. Pathologic complete response was defined as no residual invasive tumor in both the primary tumor bed and ipsilateral axillary lymph nodes (ypT0/Tis, N0) (20). An RCB class was also calculated based on pathological characteristics at surgery (9).

### Statistical Analysis

Disease-free survival (DFS) was defined as the elapsed time from the date of curative surgery to detect any recurrences, including loco-regional and distant metastases. Overall survival (OS) was defined as the duration between curative surgery and death. DFS and OS were analyzed using the Kaplan–Meier method. Differences among the groups with different characteristics were estimated using the t-test in univariate analysis. In multivariate analysis, Cox proportional-hazards regression was used to estimate hazard ratios (HRs), concordance indexes (c-indexes), and 95% confidence intervals (CIs). Statistical analyses were executed with R version 4.0.1 (R Foundation for Statistical Computing, Vienna, Austria; http://cran.r-project.org). Two-tailed p-values of <0.05 were considered statistically significant in all analyses.

### Prediction Model Development

A prediction model for DFS and OS was developed on the basis of semi-quantitative Ki-67 grade (range 1–4) and RCB class (range 1–3). We developed RPCB, which was calculated as the sum across all parameters of the Cox-coefficient for the particular parameter, multiplied by the parameter value of the patient. *b1* and *b2* were coefficients from the Cox model of RCB class and semi-quantitative Ki-67 grade.

RPCB = *b1*(RCB class) +*b2*(ln[semi-quantitative Ki67 grade + 0.1]) (21)

Performance comparison of the RPCB score system, RCB class, and Ki-67 grade was performed using Cox proportional-hazards regression. The binary logistic regression method was used for the 3-year survival prediction model development. We calculated the area under the curve (AUC) of the receiver operating characteristic (ROC) curve.

For data validation, we performed internal validation with 500 bootstrap resampling datasets (out of bag data used for testing sets).

## Results

### Clinical and Pathological Characteristics

**Figure 1:**
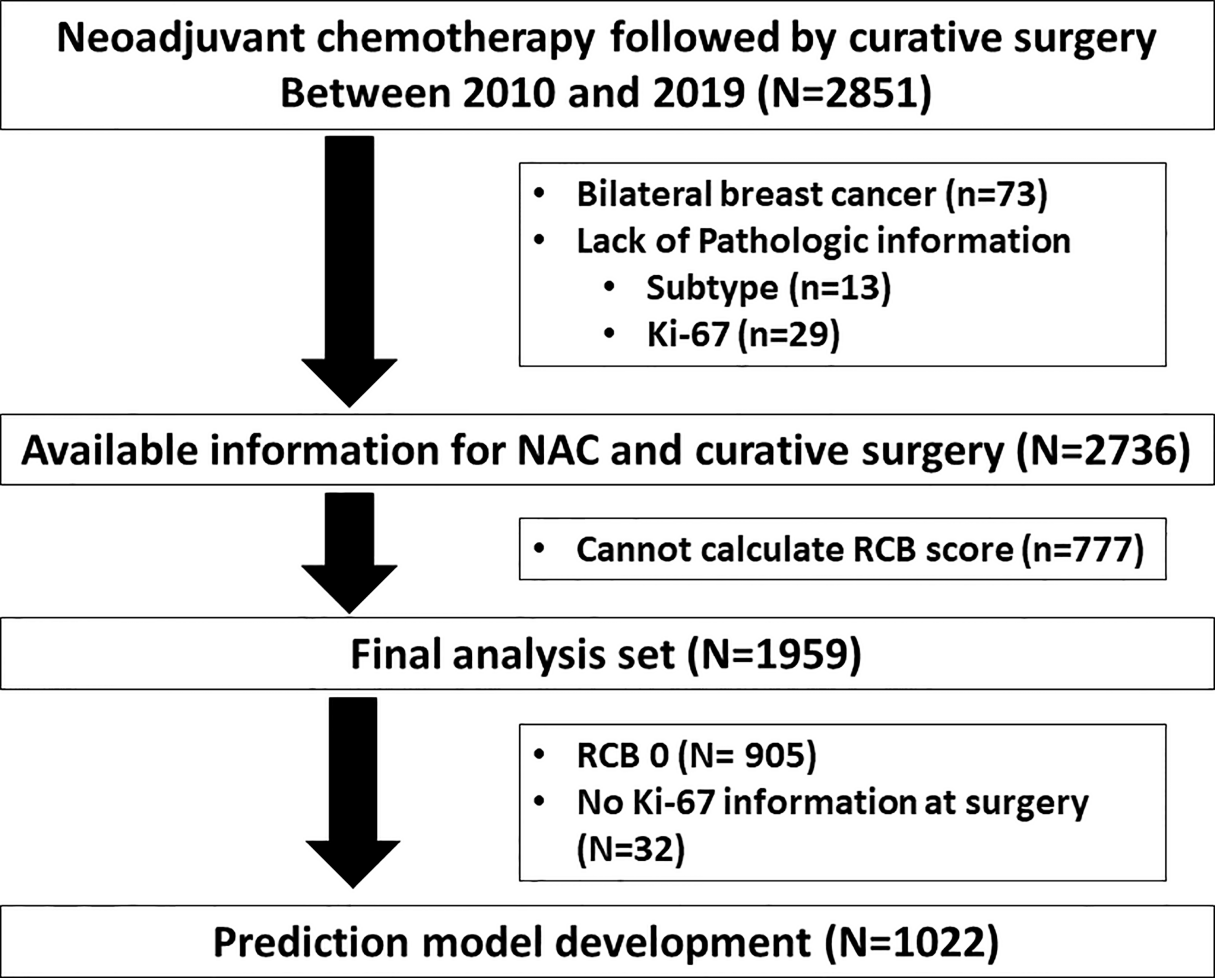
Consort diagram.

In all, 2,851 patients with BC who received NAC at the Samsung Medical Center from 2010 to 2019 were analyzed (**Figure 1**). Among 2,851 patients, 73 with bilateral BC and 42 with no pathologic information were excluded from further analysis. We further excluded 777 patients because their RCB score could not be calculated due to a lack of pathological information at the operation. Therefore, 1,959 BC patients were included in our analysis. We have described the pathological and clinical characteristics of the patients in **Supplementary Table 1**.

### Clinical Characteristics and Survival Outcome

We performed multivariate analysis to evaluate the relationship between clinical characteristics and survival outcome. We observed 220 cases of distant recurrence of BC and 81 cases of BC-related death. The median follow-up duration was 37 months (InterQuartile Range [IQR]: 18.9–48.3). Advanced clinical stage (HR of stage IIIC vs. IIA: 3.053; p <0.001), high expression of Ki-67 at curative surgery (HR of Ki-67 4+ vs. 1+: 4.080; p <0.001) and high score of the RCB class (HR of class III vs. I: 2.749; p <0.001) were associated with poor DFS (**Table 1**). In OS, high expression of Ki-67 at curative surgery (HR of Ki-67 4+ vs. 1+: 7.624, p = 0.015) and high score of RCB class (HR of class III vs. I: 2.749, p <0.001) affected to a poor survival outcome (**Supplementary Table 2**).

**Table 1:** Disease free survival prediction according to RCB class, Ki-67 and RPCB model.

Model 1		coef	Se(coef)	z	Pr(>|z|)	Type III p-value	Hazard ratio	95% CI^1^ of HR^2^		C-index	95% CI of C-index	
RCB^3^	2 vs 1	0.715	0.291	2.456	0.014	<.001	2.045	1.155	3.619	0.670	0.632	0.708
	3 vs 1	1.818	0.291	6.253	<.001		6.161	3.485	10.894			
**Model 2**		**coef**	**Se** **(coef)**	**z**	**Pr(>|z|)**	**Type III p-value**	**Hazard ratio**	**95% CI of HR**		**C-index**	**95% CI of C-index**	
Ki-67	2 vs 1	1.010	0.240	4.213	<.001	<.001	2.746	1.716	4.393	0.699	0.661	0.736
	3 vs 1	1.024	0.225	4.545	<.001		2.785	1.790	4.330			
	4 vs 1	1.511	0.192	7.855	<.001		4.530	3.107	6.603			
**Model 3**		**coef**	**Se** **(coef)**	**z**	**Pr(>|z|)**	**Type III p-value**	**Hazard ratio**	**95% CI of HR**		**C-index**	**95% CI of C-index**	
RPCB^4^-DFS^5^		1.000	0.089	11.290	<.001		2.718	2.285	3.233	0.751	0.710	0.792
RCB	2 vs 1	0.411	0.294	1.398	0.162	<.001	1.509	0.848	2.685			
	3 vs 1	1.602	0.292	5.485	<.001		4.964	2.800	8.800			
Ki-67	2 vs 1	0.927	0.240	3.860	<.001	<.001	2.526	1.578	4.043			
	3 vs 1	1.007	0.226	4.455	<.001		2.736	1.757	4.260			
	4 vs 1	1.497	0.195	7.670	<.001		4.467	3.047	6.547			

^1^Confidence interval; ^2^Hazard ratio; ^3^Residual cancer burden; ^4^Residual proliferative cancer burden; ^5^Disease free survival.

### Survival Model According to Expression of Postop. Ki-67 in RCB Class

We performed survival analysis according to Ki-67 grade in each RCB class. In DFS, postop Ki-67 at curative surgery had a prognostic value in RCB class I (p = 0.011), class II (p = 0.001), and class III (p <0.001) (**Supplementary Figures 1A–C**). In OS, postop. Ki-67 did not have prognostic value in RCB class I (p = 0.378) but did in class II (p = 0.012) and class III (p <0.001) (**Supplementary Figures 1D-–F**). These findings suggest the possibility that the addition of Ki-67 to the RCB class might improve the accuracy of prediction for DFS and OS in patients who are undergoing NAC with BC.

### Residual Proliferative Cancer Burden Model for DFS and OS

For development of the RPCB model for DFS and OS, we excluded cases of RCB 0 and those of the absence of Ki-67 value at curative surgery (**Figure 1**). In 1,022 cases, disease recurrence from BC was 170 (16.6%) and BC related death was 68 (6.7%) (**Supplementary Table 3**).

RPCB model had 2.718 in HR (95% of CI: 2.285, 3.233) and 0.751 in c-index (95% CI: 0.710, 0.792) (**Table 1**). In the RPCB model, RCB class and Ki-67 maintained their predictive capacities (HR of RCB class II vs. I: 1.509, III vs. I: 4.964, p <0.001; HR of Ki-67 4+ vs. 1+: 4.467, 3+ vs. 1+: 2.736, 2+ vs. 1+: 2.526, p <0.001). Compared with the prediction model of the RCB class and that of Ki-67, the RPCB model had superior predictive capacity (c-index of RCB model: 0.670, 95% CI: 0.632, 0.708; c-index of Ki-67: 0.699, 95% CI: 0.661, 0.736).

RPCB model for OS had 2.718 in HR (95% of CI: 2.169, 3.407) and 0.819 in c-index (95% CI: 0.755, 0.883) (**Table 2**). RPCB model had more precisely predicted OS compared with the RCB class and Ki-67 (c-index of the RCB model: 0.720, [95% of CI: 0.660, 0.779]; c-index of Ki-67: 0.750, [95% of CI: 0.695, 0.805]).

**Table 2:** Overall survival prediction according to RCB class, Ki-67 and RPCB model.

Model 1		coef	Se(coef)	z	Pr(>|z|)	Type IIIp-value	Hazard ratio	95% CI^1^ of HR^2^		C-index	95% CI of C-index	
RCB^3^	2 vs 1	1.783	0.743	2.401	0.016	<.001	5.949	1.388	25.501	0.720	0.660	0.779
	3 vs 1	3.070	0.741	4.142	<.001		21.534	5.039	92.035			
**Model 2**		**coef**	**Se** **(coef)**	**z**	**Pr(>|z|)**	**Type III** **p-value**	**Hazard ratio**	**95% CI of HR**		**C-index**	**95% CI of C-index**	
Ki-67	2 vs 1	1.848	0.484	3.818	<.001	<.001	6.345	2.458	16.382	0.750	0.695	0.805
	3 vs 1	2.297	0.442	5.194	<.001		9.949	4.181	23.675			
	4 vs 1	2.663	0.419	6.359	<.001		14.344	6.312	32.598			
**Model 3**		**coef**	**Se** **(coef)**	**z**	**Pr(>|z|)**	**Type III** **p-value**	**Hazard ratio**	**95% CI of HR**		**C-index**	**95% CI of C-index**	
RPCB^4^-OS^5^		1.000	0.115	8.677	<.001		2.718	2.169	3.407	0.819	0.755	0.883
RCB	2 vs 1	1.185	0.739	1.604	0.109	<.001	3.272	0.769	13.919			
	3 vs 1	2.575	0.733	3.511	<.001		13.133	3.119	55.300			
Ki-67	2 vs 1	1.606	0.485	3.308	0.001	<.001	4.983	1.924	12.904			
	3 vs 1	2.166	0.443	4.888	<.001		8.719	3.659	20.777			
	4 vs 1	2.579	0.422	6.115	<.001		13.184	5.768	30.135			

^1^Confidence interval; ^2^Hazard ratio; ^3^Residual cancer burden; ^4^Residual proliferative cancer burden; ^5^Overall survival.

### Three Year DFS Prediction According to RPCB Model

**Figure 2:**
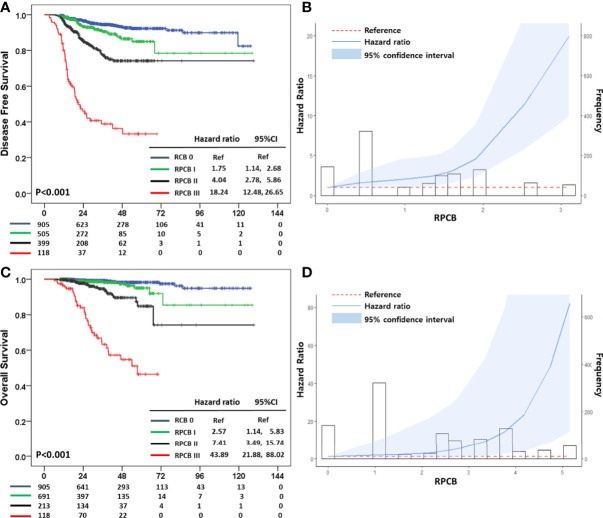
**(A)** Disease Free Survival (DFS) according to Residual Proliferative Cancer Burden (RPCB) class after neoadjuvant chemotherapy (NAC), **(B)** Hazard ratio (HR) according to RPCB score for DFS, **(C)** Overall Survival (OS) according to RPCB after NAC, **(D)** HR according to RPCB score for OS.

We divided them into three classes of the RPCB model according to their value. After making the RPCB class, we performed survival analysis in terms of 3-year DFS. In the RPCB model, there were 94.5% of 3-year DFS in RCB 0, 90.6% in RPCB class I, 77.3% in class II, and 38.9% in class III (p <0.001) (**Figures 2A-B**). HR of RPCB class I was 1.75 (95% CI: 1.14, 2.68), 4.04 of RPCB class II (95% CI: 2.78, 5.86), and 18.24 of RPCB class III (95% CI: 12.48, 26.65) compared with RCB 0.

In the RCB class model, the 3-year DFS of RCB 0 was 94.5%, 93.4% in RCB class I, 82.3% in class II, and 58.5% in class III (p <0.001) (**Supplementary Figure 2A**). HR of RCB class I vs. pCR was 1.41 (95% CI: 0.79, 2.52), 3.08 of RCB class II (95% CI: 2.14, 4.43), and 9.45 of RCB class II (95% CI: 6.58, 13.58). Additionally, Ki-67 model had 88.6% of 3-year DFS in 1+, 71.2% in 2+, 72.7% in 3+, and 61.6% in 4+ (p <0.001) (**Supplementary Figure 2B**). HR of 1.98 in the Ki-67 1+ group (95% CI: 1.33, 2.95), 5.43 of Ki-67 2+ (95% CI: 3.38, 9.71), 5.54 of Ki-67 3+ (3.55, 8.64), and 9.01 of Ki-67 4+ (95% CI: 6.15, 13.18).

In the 3-year DFS prediction model, the AUC of the RPCB model was 0.740 (95% CI: 0.691, 0.789) compared with 0.669 of the RCB model (95% CI: 0.621, 0.716), and 0.673 of Ki-67 (95% CI: 0.621, 0.726) (**Supplementary Figures 3A–C**).

We performed internal validation with 500 bootstrap resampling datasets. In DFS, the c-index of internal validation of the RPCB model was 0.751 (95% CI: 0.710, 0.792) and the AUC of the 3-year DFS model was 0.741 (95% CI: 0.692, 0.789) (**Table 3**).

**Table 3:** Internal validation of RPCB_OS and RPCB_DFS model.

	HR^1^	95% CI^2^ of HR		C-index	95% CI of C-index		AUC^3^ at 3Y^4^	95% CI of AUC	
RPCB^5^_OS^6^									
Original	2.718	2.169	3.407	0.819	0.755	0.883	0.875	0.822	0.928
Validation	2.822	1.884	3.761	0.818	0.752	0.884	0.873	0.819	0.928
RPCB_DFS^7^									
Original	2.718	2.285	3.233	0.751	0.710	0.792	0.740	0.691	0.789
Validation	2.764	2.092	3.437	0.751	0.710	0.792	0.741	0.692	0.789

^1^Hazard ratio; ^2^Confidence interval; ^3^Area under curve; ^4^Year; ^5^Residual proliferative cancer burden; ^6^Overall survival; 7: Disease free survival.

### Three Year OS Prediction According to RPCB Model

In terms of 3-year OS, RCB 0 was 99.3%, 98.6% in RPCB class I, 94.6% in class II, and 63.3% in class III (p <0.001) (**Figures 2C, D**). In OS, the HR of RPCB class I was 2.57 (95% CI: 1.14, 5.83), 7.41 of RPCB class II (95% CI: 3.49, 15.74), and 43.89 of RPCB class III (95% CI: 21.88, 88.02) compared with RCB 0.

The RCB class model had 99.4% of 3-year OS in RCB class I, 95.8% in class II, and 79.5% in class III (**Supplementary Figure 2C**). The HR of RCB I was 1.36 (95% CI: 0.38, 4.81), 6.17 of RCB II (95% CI: 3.03, 12.53), and 22.83 of RCB III (95% CI: 11.39, 45.76). According to Ki-67 grade, 98.6% in 1+, 93.9% in 2+, 86.3% in 3+, and 77.6% in 4+ (p<0.001) for 3-year OS, and we observed 1.49 of Ki-67 1+ in HR (95% CI: 0.58, 3.84), 9.42 of Ki-67 2+ (95% CI: 4.11, 21.58), 14.88 of Ki-67 3+ (95% CI: 7.11, 31.12), and 21.52 of Ki-67 4+ (95% CI: 10.85, 42.72) (**Supplementary Figure 2D**). In the 3-year OS of the RPCB model, 0.875 in AUC was observed (95% CI: 0.822, 0.928) compared with 0.747 in RCB class (95% CI: 0.684, 0.810) and 0.811 in Ki-67 grade (95% CI: 0.758, 0.862) (**Supplementary Figures 3D-F**).

Internal validation for OS presented that the c-index of internal validation was 0.818 (95% CI: 0.752, 0.884) and the AUC of the 3-year OS model was 0.873 (95% CI: 0.819, 0.928) (**Table 3**).

### RPCB Prediction Model According to BC Subtypes

**Figure 3:**
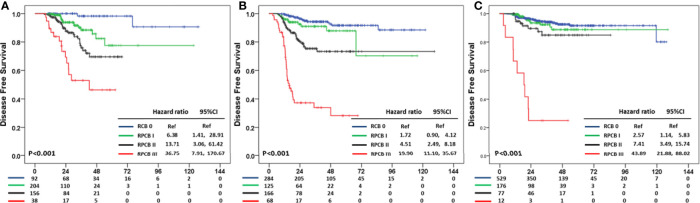
Residual Proliferative Cancer Burden prediction model according to BC subtypes in DFS **(A)** HR+HER2− subtype **(B)** TNBC subtype **(C)** HER2+ subtype.

The prediction value of the RPCB prediction model according to BC subtypes was analyzed. In DFS, RPCB class I in hormone receptor+HER2− BC had an HR of 6.38 (95% CI: 1.14, 28.91), 13.71 in class II (95% CI: 3.06, 61.42), and 36.75 in class III (95% CI: 7.91, 170.67) compared with RCB 0 (p <0.001) (**Figure 3A**). In TNBC, RPCB class I had an HR of 1.72 (95% CI: 0.90, 4.12), 4.51 in class II (95% CI: 2.49, 8.18), and 19.90 in class III (95% CI: 11.10, 35.67) compared with pCR (p <0.001) (**Figure 3B**). In HER2+ BC, HR of 2.17 in RPCB class I (95% CI: 1.14, 5.83), 7.41 of class II (95% CI: 3.49, 15.74), and 43.89 of class III (95% CI: 21.88, 88.02) (p <0.001) (**Figure 3C**).

**Figure 4:**
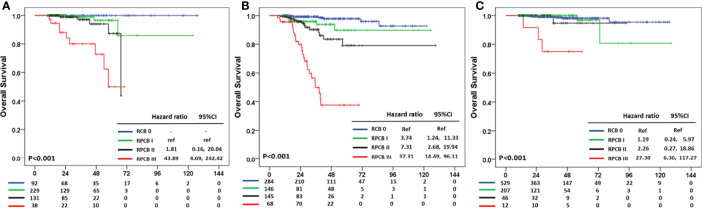
Residual Proliferative Cancer Burden prediction model according to BC subtypes in OS. **(A)** HR+HER2− subtype. **(B)** TNBC subtype. **(C)** HER2+ subtype.

In OS, the pCR group in hormone receptor +HER2− BC did not experience any death events, and therefore we calculated HR compared with RPCB class I. In hormone receptor+HER2− BC, the HR of RPCB class II was 1.81 (95% CI: 0.16, 20.04) and 43.89 of RPCB class III (95% CI: 4.69, 242.42) (p <0.001) (**Figure 4A**). The RPCB model also well predicted OS in TNBC as well as HER2+ BC. RPCB class I had HR of 3.74 (95% CI: 1.24, 11.33), 7.31 of class II (95% CI: 2.68, 19.94), and 37.31 of class III (95% CI: 14.49, 96.11) compared with pCR group (p <0.001) (**Figure 4B**). Additionally, HR was 1.19 of class I (95% CI: 0.24, 5.97), 2.26 of class II (95% CI: 0.27, 18.86), and 27.30 of class III (95% CI: 6.36, 117.27) compared with pCR group in HER2+ BC (p <0.001) (**Figure 4C**).

### Disease Free Survival and Overall Survival According to RCB Class and Post-Op Ki-67

We also evaluated DFS and OS according to RCB class and post-op Ki-67, respectively. The RCB class well predicted DFS in all BC subtypes with statistical significance (ps <0.001) (**Supplementary Figure 4**). However, we did not find the DFS difference between RCB class 0 and I in all BC subtypes. The OS predictive value of the RCB class decreased in hormone receptor+HER2− BC (p = 0.580) (**Supplementary Figure 5**). In HER+ BC, only RCB class III predicted different OS and RCB class relatively well predicted OS in TNBC (p <0.001), but RCB class I did not predict different OS compared with RCB class 0.

Post-op Ki-67 predicted DFS and OS according to BC subtypes (**Supplementary Figure 5**). Post-op Ki-67 well predicted DFS in hormone receptor +HER2− and TNBC subtypes, whereas DFS of HER2+ BC did not correlate with post-op Ki-67. OS in hormone receptor +HER2− BC and TNBC were well predicted by post-op Ki-67 (p = 0.001 and p <0.001), but only RCB class III had a different OS compared with RCB 0 in HER2+ BC.

## Discussion

We evaluated the role of Ki-67 as a prognostic value in combination with RCB class, which is the strongest prognostic factor of BC specific survival in patients who received NAC followed by curative surgery. For predicting both DFS and OS, residual proliferative cancer burden (RPCB) consisting of grade of post-op Ki-67 expression and score of RCB class had superior prognostic value compared with that of RCB class. Moreover, like the RCB class, our model of the RPCB class had a predictive value regardless of BC subtypes. Therefore, the RPCB class was easy to use and precisely predicted BC prognosis.

Ki-67 has been considered the prognostic marker and predictive marker for response to NAC (22–24). BC with high Ki-67 had a higher pCR rate but shorter survival compared with that with low Ki-67 in case of TNBC (24–26). Additionally, Ki-67 change during NAC was associated with NAC response and prognosis (24). We analyzed the prognostic value of Ki-67 expression at BC diagnosis and curative surgery after NAC and the change of Ki-67 expression during NAC in our NAC cohort. In multivariate analysis, Ki-67 at BC diagnosis did not impact on DFS and OS. Change of Ki-67 during NAC and postoperative Ki-67 at curative surgery impacted BC survival (data not shown).

During the last decade, treatment strategies for BC have been greatly updated. However, neoadjuvant chemotherapeutic regimens have not been changed greatly, except for HER2 targeting agents. In a neoadjuvant setting, doxorubicin plus cyclophosphamide followed by taxane has been the standard chemotherapeutic regimen for HER2− BC subtypes (2). In HER2+ BC, adding pertuzumab to trastuzumab has become a new standard since 2016 (2). A previous study for NAC suggested that the NAC regimen affected RCB and/or the pathologic complete response rate in BC (27, 28). But RCB was the independent prognostic factor for survival outcome regardless of the NAC regimen (29). Besides, the NAC regimen did not impact on the long-term survival outcome. Therefore, we suggested that the NAC regimen would not be associated with our RPCB prediction model.

A previous report investigating the role of Ki-67 as a prognostic factor suggested that adding Ki-67 to the RCB class improved their predictive value (21). Moreover, they incorporated other values, including ER expression and histological grade, to improve the prediction of long-term outcome. However, the benefits of adding ER expression and histological grade were not clear in terms of predicting DFS and/or OS. In our study, histological grade and BC subtype were not associated with DFS and OS, and we excluded these two factors.

In contrast to a previous study, we used semi-quantitative Ki-67 as a categorical variable, not a continuous variable. We could create our prediction model with two categorical variables; three classes of RCB and four grades of Ki-67; and an RPCB class was created based on the combination of these two categorical variables. In total, 12 scores from two categorical variables were used in our analysis, and we converted 12 scores into 3 categories of the RPCB class (**Supplementary Table 4**). This meant our RPCB prediction model would be replicable and easily adapted in a real clinic compared with a previous model (21).

Moreover, we would like to develop a prediction model that can be operated regardless of BC subtype, such as the RCB class. Each RCB class and the value of Ki-67 at curative surgery also had their own predictive values for BC prognosis in DFS and OS. However, they did not precisely predict BC prognosis in either BC subtypes or all categories. However, all categories of our model work very well in all BC subtypes of both DFS and OS.

Our model had a superior outcome in predicting OS than DFS. This trend was also observed in other prediction models, the RCB and Ki-67 models. Furthermore, the RCB class and Ki-67 models had similar predictive power in DFS, whereas the Ki-67 model had a higher value of AUC compared with the RCB model in predicting OS. This result would suggest that post-Ki-67 was more associated with DFS than OS, even though both DFS and OS were related to Ki-67. Therefore, the prediction ability of our model increased more in OS than in DFS.

In conclusion, our RPCB model, in cooperation with the anatomical RCB class and biological post-op Ki-67, more precisely predicts BC prognosis compared with the RCB class. Both DFS and OS were well estimated by our model regardless of BC subtypes. This information is helpful for decision-making regarding BC patients who had residual disease after NAC followed by curative surgery.

## Data Availability Statement

The original contributions presented in the study are included in the article/**Supplementary Material**. Further inquiries can be directed to the corresponding author.

## Ethics Statement

This study was reviewed and approved by the Institutional Review Board (IRB) of Samsung Medical Center, Seoul, Korea (IRB No. 2019-04-021) with an informed consent waiver due to the use of medical records with retrospective clinical data. This study was performed in accordance with the Declaration of Helsinki. Written informed consent for participation was not required for this study in accordance with the national legislation and the institutional requirements.

## Author Contributions

Conception/design: Y-HI and J-YK. Provision of patient data: J-YK, SL, JY, JL, SK, SN, YP, JA, and Y-HI. Collection and/or assembly of data: J-YK, JO. Statistical analysis: J-YK and KK. Manuscript writing: J-YK and Y-HI. Final approval of manuscript: J-YK, SL, JY, JL, SK, SN, YP, JA, KK, and Y-HI. All authors listed have made a substantial, direct, and intellectual contribution to the work and approved it for publication.

## Funding

This work was supported by an Institute for Information and Communications Technology Promotion grant funded by the Korean government (2018-0-00861, Intelligent SW Technology Development for Medical Data Analysis), a grant from the Korea Health Technology R&D Project through the Korea Health Industry Development Institute (KHIDI) funded by the Ministry of Health & Welfare, Republic of Korea (grant number: HR20C0025), and grants from the National Research Foundation of Korea (NRF-2017R1D1A1B03028446, NRF-2020R1F1A1072616).

## Conflict of Interest

The authors declare that the research was conducted in the absence of any commercial or financial relationships that could be construed as a potential conflict of interest.

## Publisher’s Note

All claims expressed in this article are solely those of the authors and do not necessarily represent those of their affiliated organizations, or those of the publisher, the editors and the reviewers. Any product that may be evaluated in this article, or claim that may be made by its manufacturer, is not guaranteed or endorsed by the publisher.

## Supplementary Material

Supplementary Figure 1 Disease free survival according to Ki-67 in **(A)** RCB class I, **(B)** RCB class II and **(C)** RCB class III and overall survival according to Ki-67 in **(D)** RCB class I, **(E)** RCB class II and **(F)** RCB class III.

Supplementary Figure 2 Disease free survival according to **(A)** RCB class and **(B)** Ki-67 grade and overall survival according to **(C)** RCB class and **(D)** Ki-67 grade.

Supplementary Figure 3 AUC according to 3year’s disease free survival in **(A)** RPCB class **(B)** RCB class and **(C)** Ki-67 grade and AUC according to 3 year’s overall survival in **(D)** RPCB class **(E)** RCB class and **(F)** Ki-67 grade.

Supplementary Figure 4 DFS according to RCB class in **(A)** HR+HER2- BC **(B)** TNBC **(C)** HER2+ BC and OS according to RCB class in **(D)** HR+HER2- BC **(E)** TNBC **(F)** HER2+ BC.

Supplementary Figure 5 DFS according to post op Ki-67 in **(A)** HR+HER2- BC **(B)** TNBC **(C)** HER2+ BC and OS according to post op Ki-67 in **(D)** HR+HER2- BC **(E)** TNBC **(F)** HER2+ BC.
