# Supplementary figures and images for: Improved Prediction of Survival Outcomes Using Residual Cancer Burden in Combination With Ki-67 in Breast Cancer Patients Underwent Neoadjuvant Chemotherapy
Source: Front Oncol. 2022 Jun 7;12:903372. doi: 10.3389/fonc.2022.903372 (PMC9209701; doi:10.3389/fonc.2022.903372)

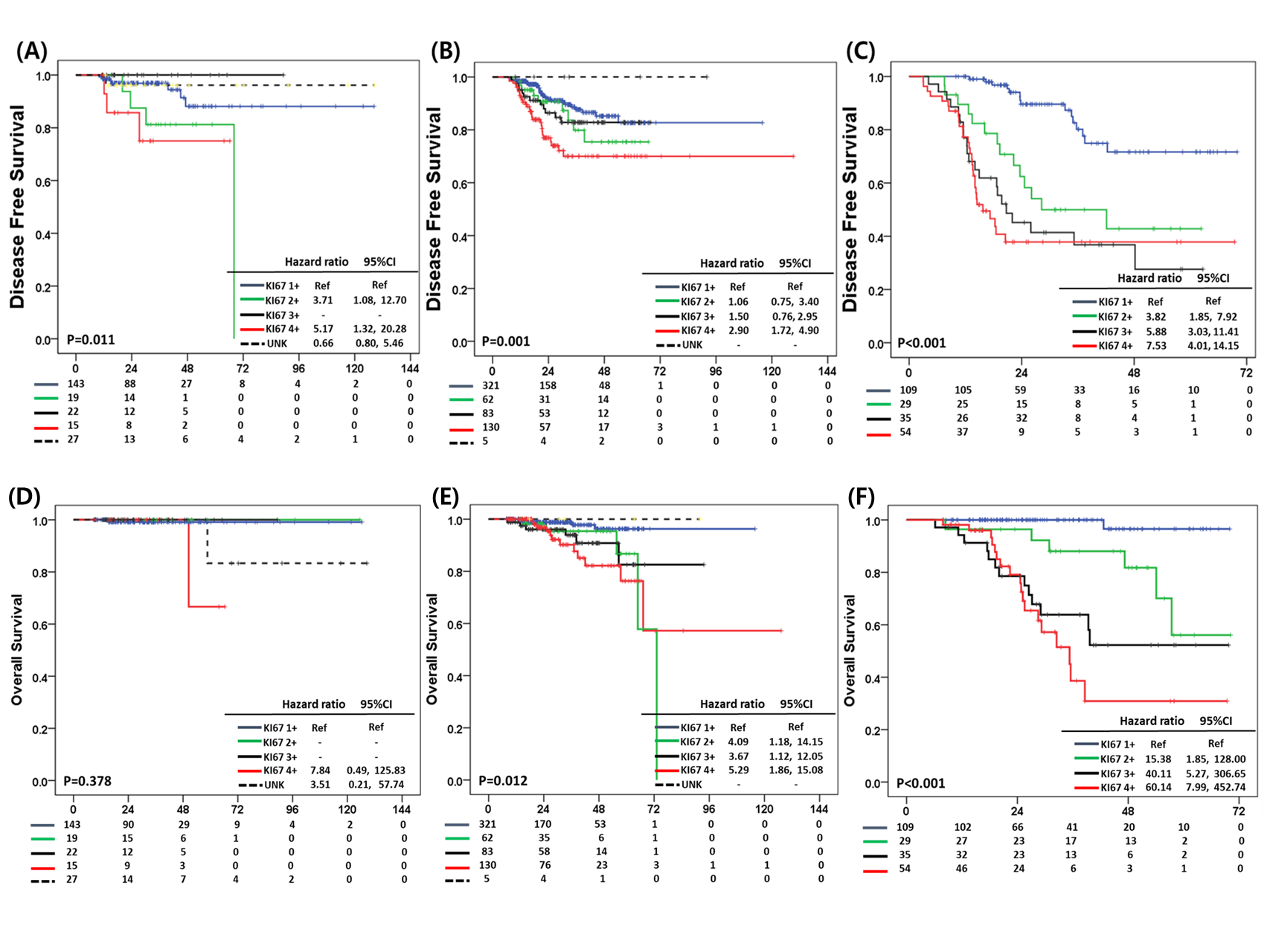

Supplement: Supplementary Figure 1 — Disease free survival according to Ki-67 in (A) RCB class I, (B) RCB class II and (C) RCB class III and overall survival according to Ki-67 in (D) RCB class I, (E) RCB class II and (F) RCB class III. [file Image_1.tif]

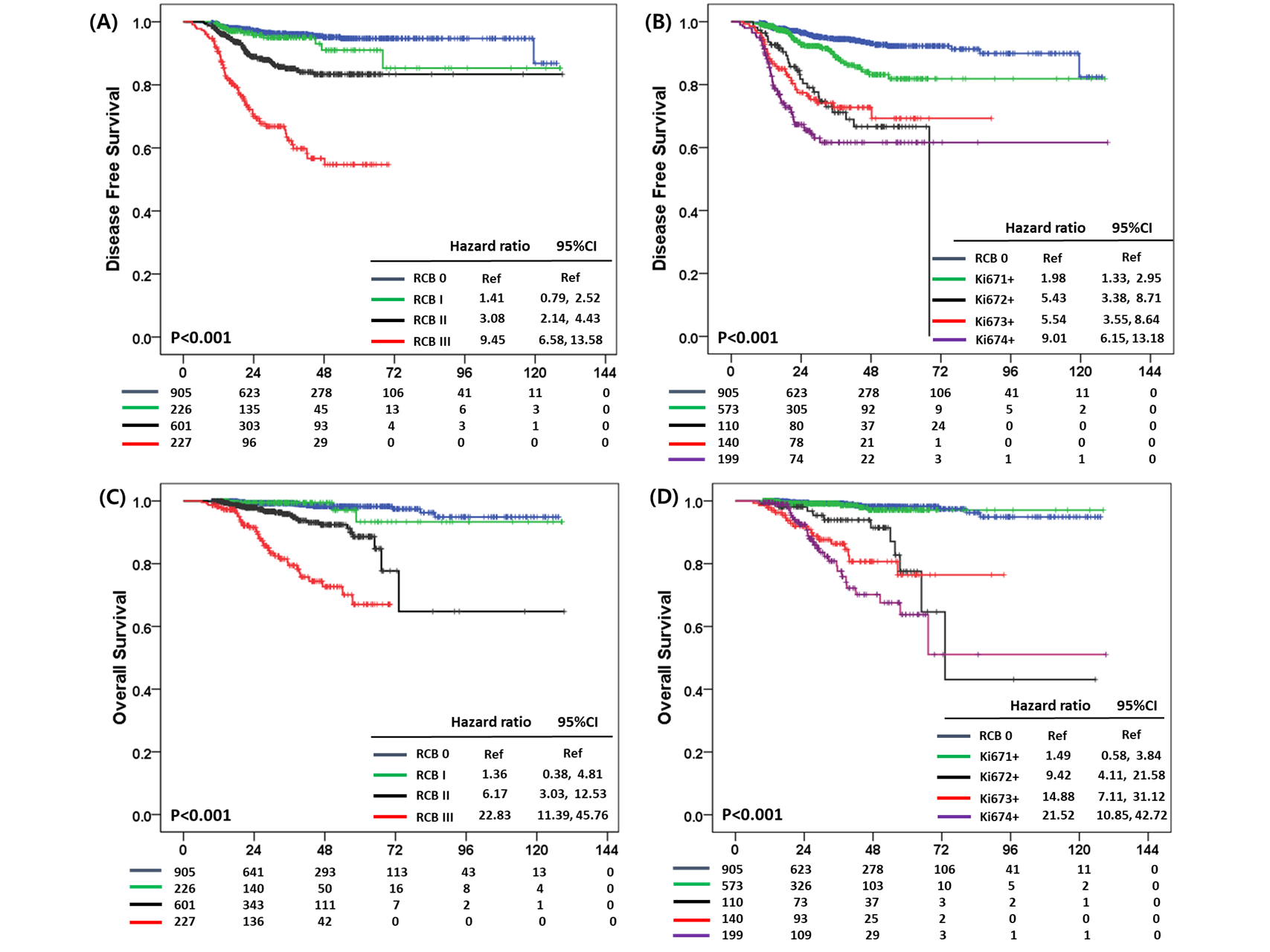

Supplement: Supplementary Figure 2 — Disease free survival according to (A) RCB class and (B) Ki-67 grade and overall survival according to (C) RCB class and (D) Ki-67 grade. [file Image_2.tif]

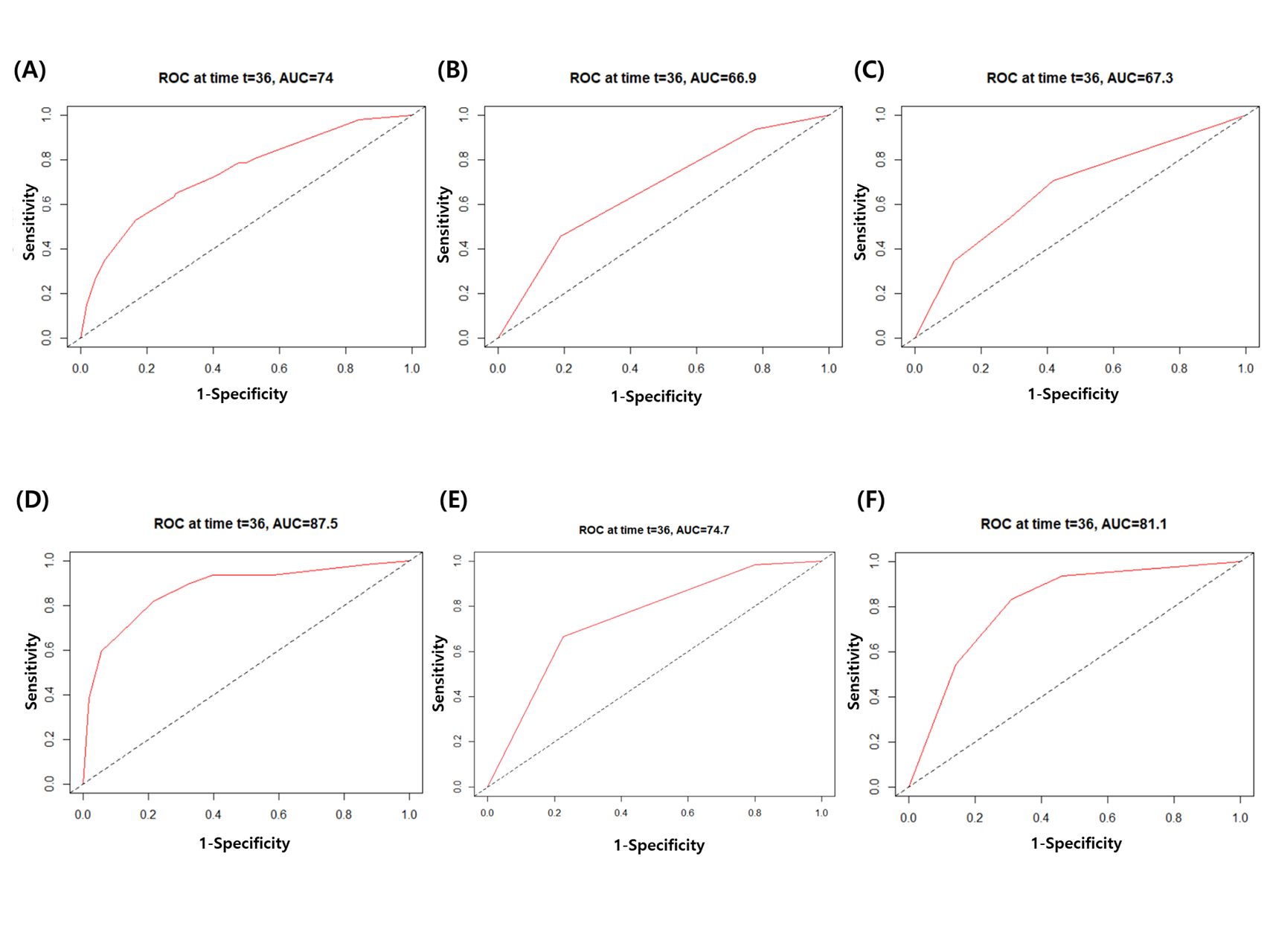

Supplement: Supplementary Figure 3 — AUC according to 3year’s disease free survival in (A) RPCB class (B) RCB class and (C) Ki-67 grade and AUC according to 3 year’s overall survival in (D) RPCB class (E) RCB class and (F) Ki-67 grade. [file Image_3.tif]

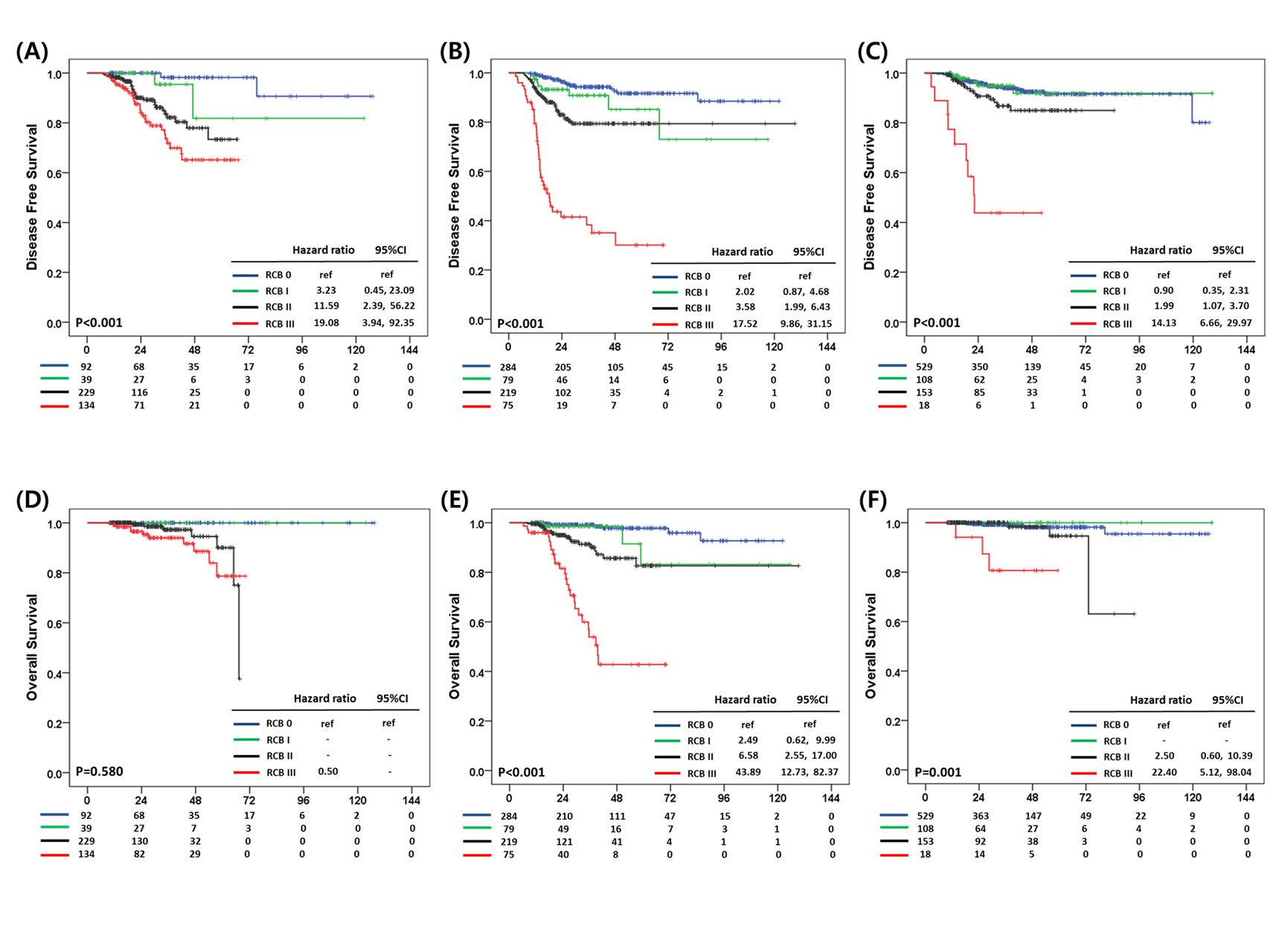

Supplement: Supplementary Figure 4 — DFS according to RCB class in (A) HR+HER2- BC (B) TNBC (C) HER2+ BC and OS according to RCB class in (D) HR+HER2- BC (E) TNBC (F) HER2+ BC. [file Image_4.tif]

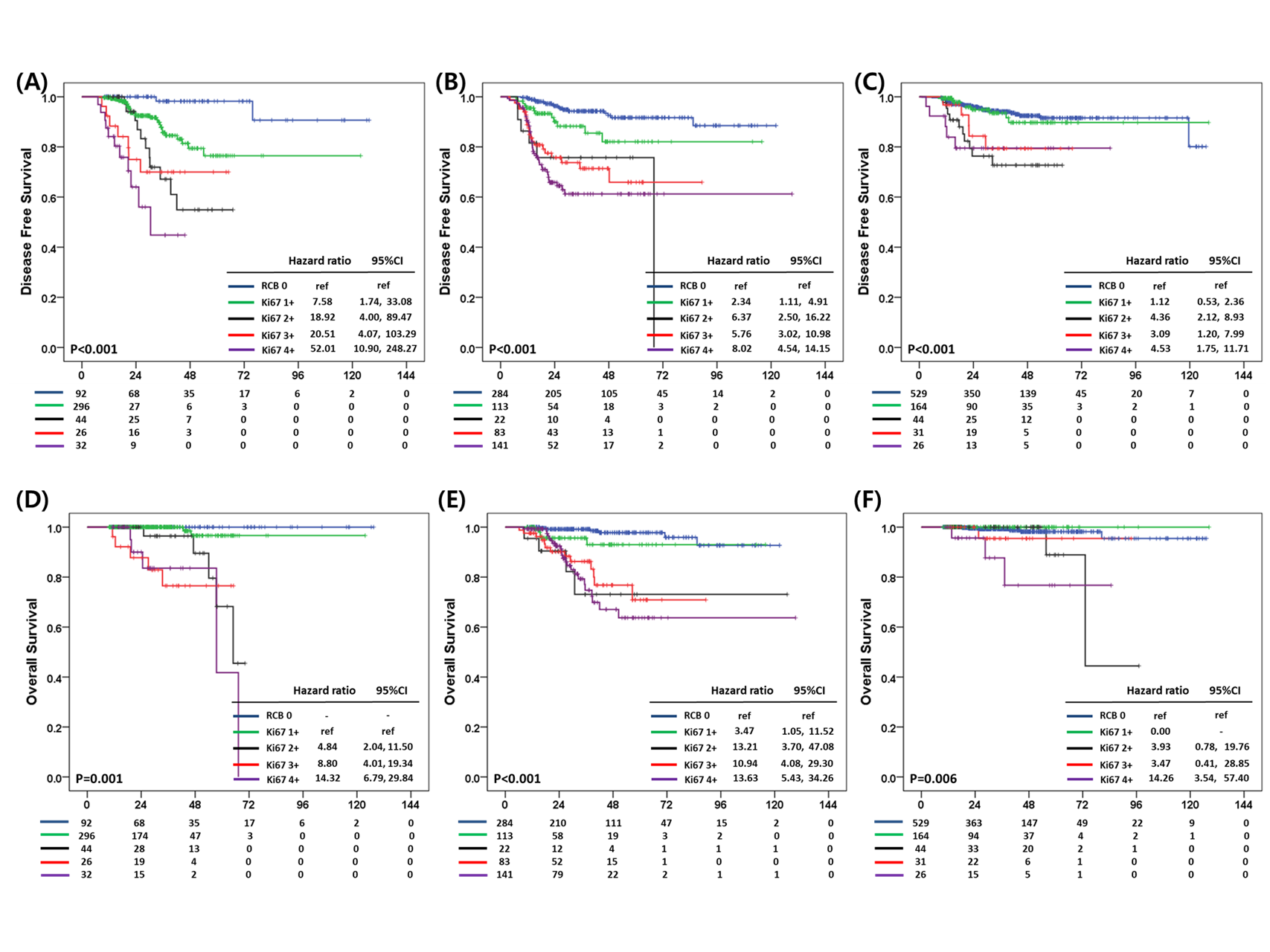

Supplement: Supplementary Figure 5 — DFS according to post op Ki-67 in (A) HR+HER2- BC (B) TNBC (C) HER2+ BC and OS according to post op Ki-67 in (D) HR+HER2- BC (E) TNBC (F) HER2+ BC. [file Image_5.tif]
