# Supplementary material for: Improved Prediction of Survival Outcomes Using Residual Cancer Burden in Combination With Ki-67 in Breast Cancer Patients Underwent Neoadjuvant Chemotherapy
Source: Front Oncol. 2022 Jun 7;12:903372. doi: 10.3389/fonc.2022.903372 (PMC9209701; doi:10.3389/fonc.2022.903372)
Supplement: Supplementary file 6 [file Table_1.docx]

**Supplementary Table 1. Multivariate analysis of clinical variables affecting to disease free survival (N=1959)**

| **Variable** | **N(%)** | **Hazard ratio** | **95% Confidence interval** | | **P-value** |
| --- | --- | --- | --- | --- | --- |
| Clinical stage |  |  |  |  | <0.001 |
| IIA | 430 (21.9) | Ref |  |  |  |
| IIB | 449 (22.9) | 0.976 | 0.553 | 1.723 |  |
| IIIA | 662 (33.8) | 1.590 | 0.969 | 2.607 |  |
| IIIB | 51 (2.6) | 1.984 | 0.865 | 4.555 |  |
| IIIC | 367 (18.7) | 3.053 | 1.843 | 5.059 |  |
| Nuclear grade at diagnosis | |  |  |  | 0.175 |
| Unknown | 413 (21.1) | Ref |  |  |  |
| I | 12 (0.6) | 1.753 | 0.000 | 9.668E+6 |  |
| II | 725 (37.0) | 2.104 | 1.164 | 11.679 |  |
| III | 1079 (55.1) | 1.904 | 1.084 | 10.431 |  |
| Histologic grade at diagnosis | |  |  |  | 0.074 |
| Unknown | 151(7.7) | Ref |  |  |  |
| I | 24 (1.2) | 0.002 | 0.000 | 2.208E+3 |  |
| II | 784 (40.0) | 0.258 | 0.090 | 0.737 |  |
| III | 1000 (51.0) | 0.301 | 0.105 | 0.859 |  |
| Subtype at diagnosis | |  |  |  | 0.340 |
| HR+^1^HER2-^2^ | 494 (25.2) | Ref |  |  |  |
| HR+HER2+ | 374 (19.1) | 1.149 | 0.631 | 2.093 |  |
| TNBC^3^ | 657 (33.5) | 1.544 | 0.919 | 2.596 |  |
| HR-HER2+ | 434 (22.2) | 1.091 | 0.585 | 2.035 |  |
| Ki-67 at diagnosis |  |  |  |  | 0.145 |
| 1+ | 366 (18.7) | Ref |  |  |  |
| 2+ | 644 (32.9) | 1.569 | 0.987 | 2.493 |  |
| 3+ | 469 (23.9) | 1.234 | 0.740 | 2.057 |  |
| 4+ | 480 (24.5) | 1.078 | 0.620 | 1.876 |  |
| Nuclear grade at curative surgery | |  |  |  | 0.844 |
| N/A^5^ | 941 (48.0) | Ref |  |  |  |
| I | 27 (1.4) | 0.014 | 0.000 | 2.179E+4 |  |
| II | 454 (23.2) | 1.416 | 0.430 | 4.662 |  |
| III | 537 (27.4) | 1.501 | 0.460 | 4.900 |  |
| Histologic grade at curative surgery | |  |  |  | 0.305 |
| I | 55 (2.8) | Ref |  |  |  |
| II | 498 (25.4) | 0.631 | 0.151 | 2.629 |  |
| III | 387 (19.8) | 0.524 | 0.111 | 2.489 |  |
| N/A^5^ | 941 (48.0) | 1.034 | 0.213 | 5.026 |  |
| Subtype at curative surgery | |  |  |  | 0.381 |
| HR+HER2- | 409 (20.9) | Ref |  |  |  |
| HR+HER2+ | 152 (7.8) | 0.655 | 0.325 | 1.319 |  |
| TNBC | 361 (18.4) | 1.116 | 0.633 | 1.967 |  |
| HR-HER2+ | 103 (5.3) | 1.546 | 0.739 | 3.235 |  |
| N/A^5^ | 934 (47.7) | 273.937 | 0.000 | 2.525E+32 |  |
| Ki-67 at curative surgery | |  |  |  | <0.001 |
| 1+ | 573 (29.2) | Ref |  |  |  |
| 2+ | 110 (5.6) | 1.900 | 1.112 | 3.248 |  |
| 3+ | 140 (7.1) | 2.251 | 1.242 | 4.079 |  |
| 4+ | 199 (10.2) | 4.080 | 2.201 | 7.563 |  |
| N/A^5^ | 937 (47.8) | 0.002 | 0.000 | 1.521E+27 |  |
| RCB^4^ class |  |  |  |  | <0.001 |
| 0 | 905 (46.2) | Ref |  |  |  |
| 1 | 226 (11.5) | 0.482 | 0.057 | 4.100 |  |
| 2 | 601 (30.7) | 0.860 | 0.099 | 7.445 |  |
| 3 | 227 (11.6) | 2.749 | 0.307 | 24.610 |  |

1: Hormone receptor; 2: Human epidermal growth factor receptor 2; 3:Triple negative breast cancer 4: Residual cancer burden; 5:Not accessed due to not enough residual tumor tissue

**Supplementary Table 2. Multivariate analysis of clinical variables affecting to overall survival (N=1959)**

| **Variable** | **N(%)** | **Hazard ratio** | **95% Confidence interval** | | **P-value** |
| --- | --- | --- | --- | --- | --- |
| Clinical stage |  |  |  |  | 0.172 |
| IIA | 430 (21.9) | Ref |  |  |  |
| IIB | 449 (22.9) | 1.139 | 0.413 | 3.142 |  |
| IIIA | 662 (33.8) | 1.527 | 0.609 | 3.831 |  |
| IIIB | 51 (2.6) | 1.832 | 0.433 | 7.753 |  |
| IIIC | 367 (18.7) | 2.426 | 0.946 | 6.219 |  |
| Nuclear grade at diagnosis | |  |  |  | 0.343 |
| N/A^5^ | 413 (21.1) | Ref |  |  |  |
| I | 12 (0.6) | 0.461 | 0.000 | 5.777E+13 |  |
| II | 725 (37.0) | 4.227 | 0.461 | 38.780 |  |
| III | 1079 (55.1) | 2.072 | 0.234 | 18.349 |  |
| Histologic grade at diagnosis | |  |  |  | 0.603 |
| N/A^5^ | 151(7.7) | Ref |  |  |  |
| I | 24 (1.2) | 0.009 | 0.000 | 6.228E+10 |  |
| II | 784 (40.0) | 0.239 | 0.029 | 1.947 |  |
| III | 1000 (51.0) | 0.287 | 0.036 | 2.296 |  |
| Subtype at diagnosis | |  |  |  | 0.592 |
| HR+^1^HER2-^2^ | 494 (25.2) | Ref |  |  |  |
| HR+HER2+ | 374 (19.1) | 0.523 | 0.127 | 2.160 |  |
| TNBC^3^ | 657 (33.5) | 1.106 | 0.470 | 2.603 |  |
| HR-HER2+ | 434 (22.2) | 0.615 | 0.188 | 2.003 |  |
| Ki-67 at diagnosis |  |  |  |  | 0.737 |
| 1+ | 366 (18.7) | Ref |  |  |  |
| 2+ | 644 (32.9) | 1.599 | 0.602 | 4.249 |  |
| 3+ | 469 (23.9) | 1.647 | 0.615 | 4.409 |  |
| 4+ | 480 (24.5) | 1.392 | 0.490 | 3.956 |  |
| Nuclear grade at curative surgery | |  |  |  | 0.921 |
| N/A^5^ | 941 (48.0) | Ref |  |  |  |
| I | 27 (1.4) | 0.009 | 0.000 | 6.743E+12 |  |
| II | 454 (23.2) | 0.607 | 0.119 | 3.091 |  |
| III | 537 (27.4) | 0.767 | 0.156 | 3.772 |  |
| Histologic grade at curative surgery | |  |  |  | 0.366 |
| N/A^5^ | 941 (48.0) | Ref |  |  |  |
| I | 55 (2.8) | 0.016 | 0.000 | 8.445E+8 |  |
| II | 498 (25.4) | 0.326 | 0.085 | 1.248 |  |
| III | 387 (19.8) | 0.361 | 0.102 | 1.272 |  |
| Subtype at curative surgery | |  |  |  | 0.197 |
| HR+HER2- | 409 (20.9) | Ref |  |  |  |
| HR+HER2+ | 152 (7.8) | 0.157 | 0.017 | 1.474 |  |
| TNBC | 361 (18.4) | 2.059 | 0.777 | 5.457 |  |
| HR-HER2+ | 103 (5.3) | 1.700 | 0.401 | 7.199 |  |
| N/A^5^ | 934 (47.7) | 1770.136 | 0.000 | 3.540E+62 |  |
| Ki-67 at curative surgery | |  |  |  | 0.015 |
| 1+ | 573 (29.2) | Ref |  |  |  |
| 2+ | 110 (5.6) | 3.631 | 1.247 | 10.575 |  |
| 3+ | 140 (7.1) | 5.659 | 1.892 | 16.924 |  |
| 4+ | 199 (10.2) | 7.624 | 2.434 | 23.885 |  |
| N/A^5^ | 937 (47.8) | 0.001 | 0.000 | 1.712E+56 |  |
| RCB^4^ class |  |  |  |  | <0.001 |
| 0 | 905 (46.2) | Ref |  |  |  |
| 1 | 226 (11.5) | 1.454 | 0.128 | 16.502 |  |
| 2 | 601 (30.7) | 5.830 | 0.536 | 63.364 |  |
| 3 | 227 (11.6) | 22.469 | 1.917 | 263.337 |  |

1: Hormone receptor; 2: Human epidermal growth factor receptor 2; 3:Triple negative breast cancer 4: Residual cancer burden; 5:Not accessed due to not enough residual tumor tissue

**Supplementary Table 3. Clinical outcome of Residual proliferative cancer burden model**

|  |  | **Death** | | **Recurrence** | |
| --- | --- | --- | --- | --- | --- |
|  | **Total** | **No** | **Yes** | **No** | **Yes** |
| **n** | **1022 (%)** | **954 (%)** | **68 (%)** | **852 (%)** | **170 (%)** |
| RCB^1^ (%) |  |  |  |  |  |
| 1 | 199 (19.5) | 197 (20.6) | 2 ( 2.9) | 185 (21.7) | 14 ( 8.2) |
| 2 | 596 (58.3) | 568 (59.5) | 28 (41.2) | 519 (60.9) | 77 (45.3) |
| 3 | 227 (22.2) | 189 (19.8) | 38 (55.9) | 148 (17.4) | 79 (46.5) |
| Ki-67 (%) |  |  |  |  |  |
| 1 | 573 (56.1) | 566 (59.3) | 7 (10.3) | 524 (61.5) | 49 (28.8) |
| 2 | 110 (10.8) | 99 (10.4) | 11 (16.2) | 83 ( 9.7) | 27 (15.9) |
| 3 | 140 (13.7) | 121 (12.7) | 19 (27.9) | 107 (12.6) | 33 (19.4) |
| 4 | 199 (19.5) | 168 (17.6) | 31 (45.6) | 138 (16.2) | 61 (35.9) |

**1: Residual Cancer Burden Class**

**Supplementary Table 4. Score board of RPCB prediction model for overall survival and disease free survival**

| **Score system** | | **RPCB_OS** | **Score system** | | **RPCB_DFS** |
| --- | --- | --- | --- | --- | --- |
| **OS** | **level** | **score** | **DFS** | **level** | **score** |
| **RCB** | **1** | **0** | **RCB** | **1** | **0** |
|  | **2** | **2** |  | **2** | **1** |
|  | **3** | **5** |  | **3** | **3** |
| **Ki-67** | **1** | **0** | **Ki-67** | **1** | **0** |
|  | **2** | **3** |  | **2** | **2** |
|  | **3** | **4** |  | **3** | **2** |
|  | **4** | **5** |  | **4** | **3** |
| **Score** | **min** | **0** | **Score** | **min** | **0** |
|  | **max** | **10** |  | **max** | **6** |
| **Score matching table** | | **0** | **Score matching table** | | **0** |
|  |  | **2** |  |  | **1** |
|  |  | **3** |  |  | **2** |
|  |  | **4** |  |  | **2** |
|  |  | **5** |  |  | **3** |
|  |  | **5** |  |  | **3** |
|  |  | **5** |  |  | **3** |
|  |  | **6** |  |  | **3** |
|  |  | **7** |  |  | **4** |
|  |  | **8** |  |  | **5** |
|  |  | **9** |  |  | **5** |
|  |  | **10** |  |  | **6** |
| **Categorization** | **1** | **0-5** | **Categorization** | **1** | **0-2** |
|  | **2** | **6-7** |  | **2** | **2-4** |
|  | **3** | **8 and more** |  | **3** | **5 and more** |
